# Correction: Mapping the dynamics of force transduction at cell–cell junctions of epithelial clusters

**DOI:** 10.7554/eLife.06656

**Published:** 2015-02-02

**Authors:** Mei Rosa Ng, Achim Besser, Joan S Brugge, Gaudenz Danuser

Ng MR, Besser A, Brugge JS, Danuser G. 2014. Mapping the dynamics of force transduction at cell–cell junctions of epithelial clusters. *eLife* **3**:e03282. doi: 10.7554/eLife.03282

Published 5 December 2014

Due to a production error, in the final published article the abstract was given as:

The integrated quantification of spontaneous dynamic cell–cell force transmissions at both multi-cellular and sub-cellular scales enables spatiotemporal correlations of stress distribution with biomolecules in small cell clusters.

This text was the Impact statement for the article. The abstract should have read:

Force transduction at cell–cell adhesions regulates tissue development, maintenance and adaptation. We developed computational and experimental approaches to quantify, with both sub-cellular and multi-cellular resolution, the dynamics of force transmission in cell clusters. Applying this technology to spontaneously-forming adherent epithelial cell clusters, we found that basal force fluctuations were coupled to E-cadherin localization at the level of individual cell–cell junctions. At the multi-cellular scale, cell–cell force exchange depended on the cell position within a cluster, and was adaptive to reconfigurations due to cell divisions or positional rearrangements. Importantly, force transmission through a cell required coordinated modulation of cell-matrix adhesion and actomyosin contractility in the cell and its neighbors. These data provide insights into mechanisms that could control mechanical stress homeostasis in dynamic epithelial tissues, and highlight our methods as a resource for the study of mechanotransduction in cell–cell adhesions.

The article has now been corrected.

