# Supplementary material for: Mapping the dynamics of force transduction at cell–cell junctions of epithelial clusters
Source: eLife. 2014 Dec 5;3:e03282. doi: 10.7554/eLife.03282 (PMC4300730; doi:10.7554/eLife.03282)
Supplement: Source code 1. — DOI: http://dx.doi.org/10.7554/eLife.03282.024 [file elife03282s001.zip › eLife03282_cellCellForceAnalysis_codes/NgBesserEtAl_eLife.03282_scripts_readme.docx]

**eLife.03282**

**Mapping the dynamics of force transduction at cell-cell junctions of epithelial clusters**

Ng MR*, Besser A*, Brugge JS, Danuser G.

Published online December 5, 2014

DOI: http://dx.doi.org/10.7554/eLife.03282

**MATLAB analysis codes accompanying manuscript**

We have included with the manuscript the core MATLAB scripts that were used to analyze our data, in order to encourage the community to adapt our cell-cell force measurement approach for future studies. Please understand that our raw codes, as they are currently written and run, are the natural product of a project that has developed, grown and organically evolved over several years as the ideas matured and more data were collected. As such, the code is in a state that is adaptable by those adept at deciphering MATLAB routines, but in no way meant to be comprehensive and directly executable by the general readership. Converting this organic code progression into a stable software will take several months of software redesign that go beyond the scope of the paper.

The core MATLAB programs included are:

calcElEnergies.m and its dependent functions, for calculating strain energies of the cell cluster (see “Cell strain energy” in the Methods section),

cutOutForceFieldManyCells.m and its dependent functions, for segmentation of cell clusters and cell-cell junctions (see “Image segmentation of cell boundaries and cell-cell interface” in the Methods section),

and clusterAnalysis.m and its dependent functions, for tracking of cells and cell-cell junctions (see “Tracking of cells and cell-cell interfaces” in the Methods section), and for calculation of cell-cell forces and stresses using the force-balancing and thin-plate FEM approaches (see “Finite element method for cell-cell force measurements” in the Methods section).

The MATLAB toolboxes required for execution of the included programs are:

Image Processing Toolbox,

Partial Differential Equation Toolbox,

Symbolic Math Toolbox,

and Statistics Toolbox.

The last MATLAB version the analysis codes were tested is MATLAB R2012b.

As an example of the inputs required for the above analysis programs, we have also included sample data files forceField.mat (required for cutOutForceFieldManyCells.m, calcElEnergies.m and clusterAnalysis.m), displField.mat (required for calcElEnergies.m) and several E-cadherin-GFP fluorescent images (required for cutOutForceFieldManyCells.m).

forceField.mat and displField.mat are data files that should be generated by the end of traction force microscopy calculations, which is the first step of our cell-cell force measurement method. At the point of publication the software bundle does not include methods for traction force calculation. However, a user-friendly and mathematically advanced software package for this purpose is under review (revision) elsewhere. It will be released as soon as this manuscript is accepted. It will be downloadable from our website lccb.hms.harvard.edu and contain among several options the bead tracking and Fourier Transform Traction Cytometry (FTTC) methods used for the here described work. Please make sure to regularly visit this website for updates of our software packages.

All other approaches used for our analysis are detailed in the Methods section, such that those who wish to reproduce the results can do so.

We hope our methodology and the results we have reported in this article will foster further interests and stimulate new hypotheses and studies on cell biomechanics and mechanotransduction.
