# Supplementary figures and images for: Mapping the dynamics of force transduction at cell–cell junctions of epithelial clusters
Source: eLife. 2014 Dec 5;3:e03282. doi: 10.7554/eLife.03282 (PMC4300730; doi:10.7554/eLife.03282)

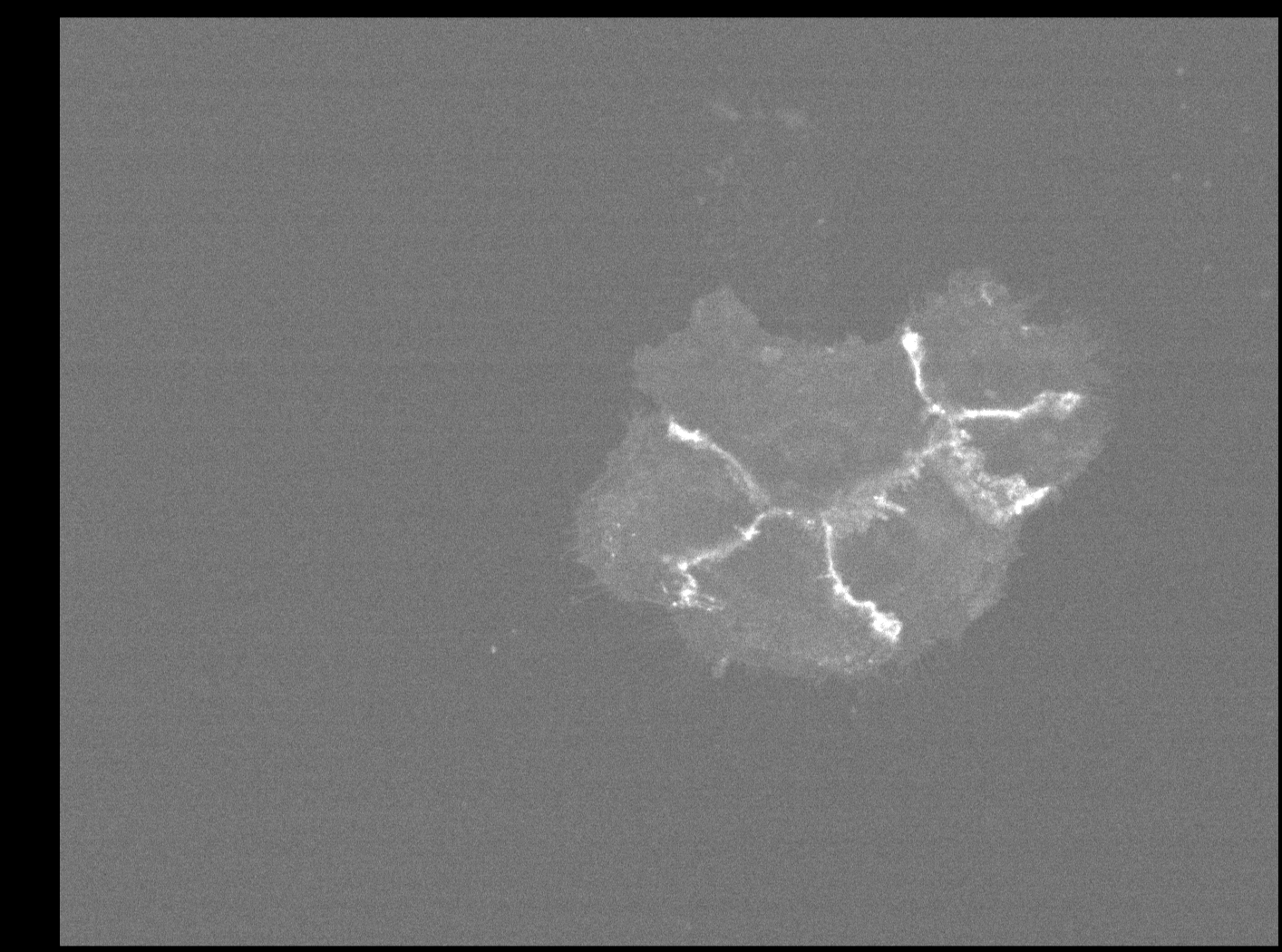

Supplement: Source code 1. — DOI: http://dx.doi.org/10.7554/eLife.03282.024 [file elife03282s001.zip › eLife03282_cellCellForceAnalysis_codes/sampleData/data/6EcadFinal/registered_registered_2010_09_23_TFM__w3491 with shutter_s12_t169.tif]

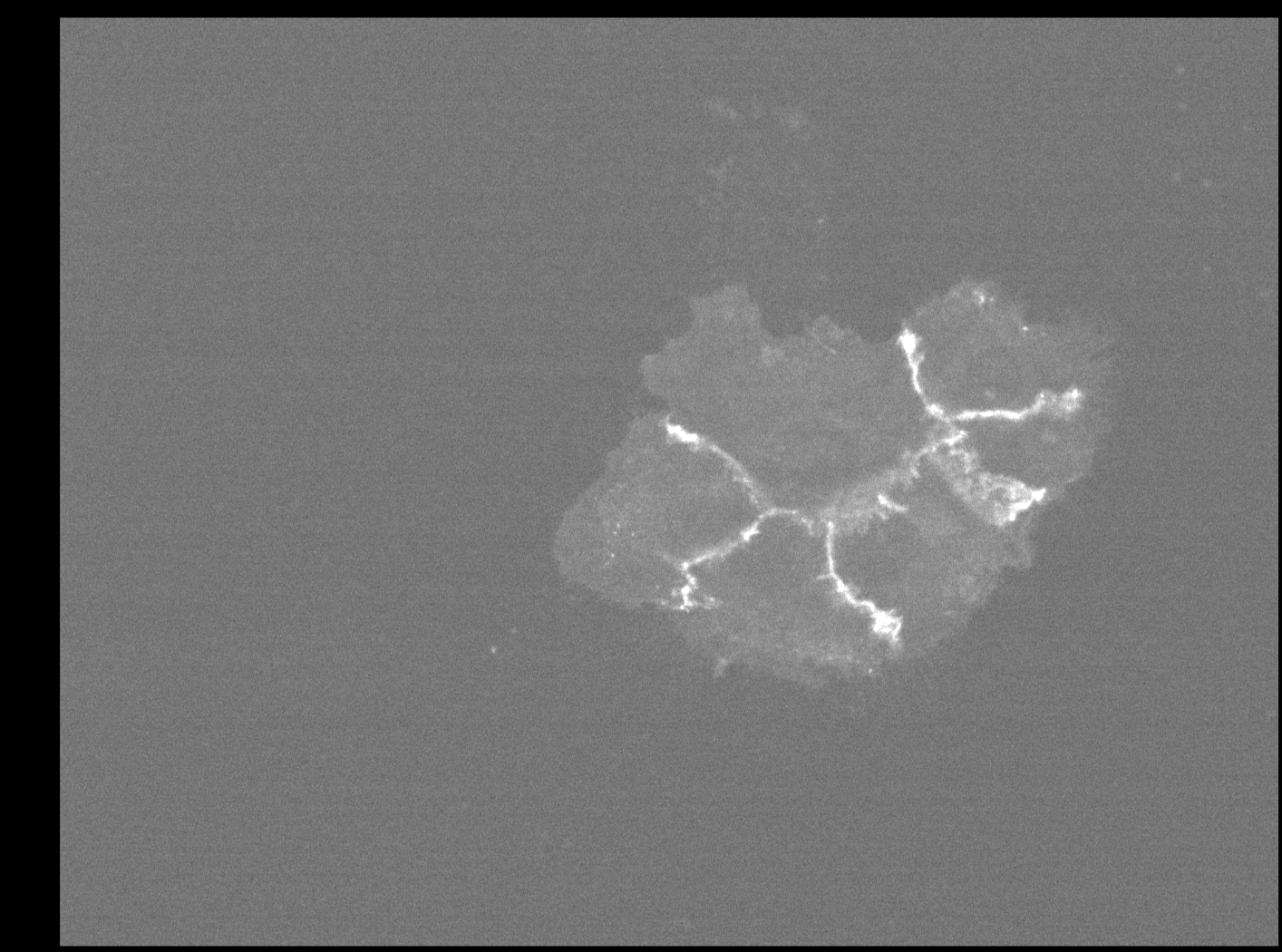

Supplement: Source code 1. — DOI: http://dx.doi.org/10.7554/eLife.03282.024 [file elife03282s001.zip › eLife03282_cellCellForceAnalysis_codes/sampleData/data/6EcadFinal/registered_registered_2010_09_23_TFM__w3491 with shutter_s12_t170.tif]
